# Formulation of Levocetirizine-Loaded Core–Shell Type Nanofibrous Orally Dissolving Webs as a Potential Alternative for Immediate Release Dosage Forms

**DOI:** 10.3390/pharmaceutics14071442

**Published:** 2022-07-11

**Authors:** Adrienn Kazsoki, Barnabás Palcsó, Safaa Mohammed Omer, Zoltan Kovacs, Romána Zelkó

**Affiliations:** 1 University Pharmacy Department of Pharmacy Administration, Semmelweis University, Hőgyes Endre utca 7-9, H-1092 Budapest, Hungary; kazsoki.adrienn@pharma.semmelweis-univ.hu (A.K.); palcso.barnabas@pharma.semmelweis-univ.hu (B.P.); safopharma11@gmail.com (S.M.O.); 2 Department of Measurements and Process Control, Institute of Food Science and Technology, Hungarian University of Agriculture and Life Sciences, Somlói Street 14-16, H-1118 Budapest, Hungary; kovacs.zoltan.food@uni-mate.hu

**Keywords:** nanofiber, core–shell fiber, electrospinning, antiallergic formulation, intraoral drug delivery system, solid-state characterization, in vitro dissolution study, taste masking excipients, e-tongue

## Abstract

Several applications of nanofiber-based systems are based on their corresponding functionality-related properties, which often cannot be satisfied by a fiber web with a monolithic structure because of the various physicochemical properties and amounts of embedded compounds. Therefore, one of the main directions in the development of fiber systems is creating core–shell type complex fiber structures that can provide application-specific properties to the fiber matrix. The present study aimed to formulate levocetirizine-loaded core–shell type hydrophilic polymer-based fibrous systems. The core phase contained the antihistamine levocetirizine, while the permeation enhancer (Na-taurocholate), the local pH regulator (citric acid), and the cyclodextrin used as a taste masking agent were included in the shell phase of the fibrous formulation. Scanning electron microscopy images indicated that a randomly oriented homogeneous fibrous structure was obtained, while the Raman mapping and chemometric analysis confirmed the partially formed core–shell structure. A fast release rate of the antihistamine drug from the complex structural fibrous system was obtained (within 1 min complete dissolution can be observed) due to its increased surface area to volume ratio and its more favorable wettability properties, which consequently allows for more erosion. The masking properties against the unpleasant bitter taste of API of the formulated complex nanostructure were confirmed by the results of the electronic tongue. The formulated complex nanostructure enabled fast and complete release of the API, providing a potential enhancement in the rate and extent of absorption while masking the unpleasant taste of levocetirizine, which has a high impact on the patient adherence. All in all, the results show that the developed orally dissolving fibrous web formulation can be a potential alternative to the commercially available orally disintegrating tablets.

## 1. Introduction

The biomedical applications of nanofiber systems have become an area of intensive research in recent years, which can be traced back to their unique properties, e.g., high surface area to volume ratio, high porosity, and their structure being able to mimic the extracellular matrix [1,2]. Among other things, they can offer a solution for the formulation of active pharmaceutical ingredients with unfavorable physicochemical properties, so their study has many possibilities from a pharmaceutical point of view [3,4,5,6,7]. The use of nanofibrous systems to formulate various dosage forms has recently come to the forefront of pharmaceutical technology research [8]. The diversity of the structure and the chemical quality of the polymer allow for targeted [9] or controlled drug delivery and protect the drug from premature biodegradation and polymer erosion. Vass et al. reported promising results for the future positioning of the technique. In their work, they successfully applied the high-speed electrostatic process as an efficient and scalable alternative to freeze-drying for the production of an intravenously administrable reconstitution powder-based drug formulation [10].

Nanofibrous systems in a variety of forms can be used in several administration routes. Among others, nanofibrous drug delivery systems can be used in the preparation of various intraoral (buccal and sublingual) dosage forms, which have several advantageous properties [11,12,13]. Due to the anatomical features of the oral cavity and the buccal mucosa, both local and systemic effects can be achieved with intraoral administration [11,14]. The compound administered in this way avoids gastric acid exposure and first-pass metabolism in the liver, thereby increasing the bioavailability of the drug. An immediate effect can be achieved using hydrophilic polymer-based nanofibrous sheets, which allows essential and liquid-free medication intake in acute cases. This formulation is also suitable for patients with poor cooperation and difficulty in swallowing [15]. A recently published work [16] demonstrated the better performance of the prednisolone sodium phosphate-loaded electrospun nanofibers compared to the orally disintegrating films (ODFs), particularly in the disintegration and dissolution studies. Although the encapsulation efficiency of the ODFs was higher than that of the nanofibers, the disintegration time was substantially quicker for the electrospun nanofibers than for the ODFs [16].

The versatility of a nanofibrous system is based on its functionality-related properties, which often cannot be reached by a fiber mat with a monolithic structure. One of the main directions in developing fibrous systems is to create a multicomponent fibrous structure that can provide application-relevant unique properties to the fibrous matrix and allow the formulation to meet enhanced quality and functional requirements.

A complex composite fibrous structure can be fabricated from monolithic fibers. Liu et al. categorized these systems into three groups according to the process [17]:

1. Utilize the electrospun nanofibers directly or as the reinforcing agents, in which the unique properties of the fibers are exploited to endow the composites with improved functional performances [17].
2. Assemble the electrospun nanofibers into a designed macrostructure, by layer-by-layer deposition, self-assembly, and using a 3D collection template to fold and stack 2D nanofiber mats [17].
3. Create composite structures from monolithic nanocomposites within the fiber, which can be feasible by the multifluid electrospinning technique in one step [17].

Among the many possible structures, the core–shell and Janus structures are the most fundamental of the multiple-chamber structures for developing novel sorts of functional materials [18]. These processes and structures can provide a promising platform for developing new kinds of medicated materials [9,17,19,20,21,22].

These systems can be used for separate embedding of multiple active ingredients, formulation of environmentally sensitive substances, and modification of the release profile of active ingredients embedded in fibers. Such are formulations used to achieve controlled release. The latter has been extensively studied in the literature [19,23,24,25]. However, in the present work, a novel approach is taken.

In the intraoral dosage form, a requirement is to increase the permeability required by the moderate permeability of the buccal mucosa and the taste masking aimed at achieving adequate adherence [26,27]. One of the disadvantages of a fiber formulation with a monolithic structure is that it is inapplicable for embedding large amounts of active ingredients, but core–shell type fibrous systems provide a promising means to overcome this obstacle [24,28,29,30]. The excipients can be embedded into the different layers of the core–shell fibers, and the system can ensure both adequate properties and the right manufacturability.

Therefore, the present research aims to formulate a core–shell type nanofibrous system based on a hydrophilic polymer containing an antihistamine (levocetirizine) model drug by coaxial electrospinning. Another purpose is to ensure an immediate effect (rapid release and absorption) of the intraoral dosage form. In order to achieve proper absorption, we also investigate the effect of a local pH modifier (which increases the neutral form of the active ingredient in the precursor solution used for fiber formation) and permeability-enhancing excipients (e.g., polysorbate, cyclodextrin derivatives).

## 2. Materials and Methods

### 2.1. Materials

Levocetirizine dihydrochloride was provided by Egis Pharmaceuticals Ltd. (Budapest, Hungary). The polyvinyl alcohol (PVA, Mowiol^®^ 18–88, average molecular weight Mw~130,000 g mol^−1^) and polyvinylpyrrolidone (PVP, Kollidon K90, average molecular weight, Mw~1,500,000 g mol^−1^) used to prepare the precursor solutions for fiber formation were products of Sigma Aldrich Ltd. (Budapest, Hungary). Hydroxypropyl-β-cyclodextrin (average degree of substitution *(n*): 4.5, average molecular weight: 1135.0 + *n* × 58.1 g mol^−1^) was purchased from Cyclolab Ltd. (Budapest, Hungary). Polysorbate 80 and citric acid were supplied by Molar Chemicals (Budapest, Hungary). Sodium taurocholate was the product of TCI Ltd. (Tokyo, Japan). Viscous solutions were prepared using distilled water and ethanol (96%, Molar Chemicals, Hungary) of pharmacopeial grade without further purification.

### 2.2. Fiber Formation Experiments

#### 2.2.1. Preparation of Viscous Polymer Precursor Solutions for Electrospinning

The formulation was based on water-soluble biocompatible polymers. The core part of the formulation was PVA, while the shell part was PVP. In the case of the composite fiber system, the core phase contained the antihistamine. For the active ingredient, all precursor solutions were 3% *w*/*w*. In order to reduce the unpleasant taste of the drug, HP-β-CD was used in different molar ratios (drug/CD = 1:1 (*n:n*), 1:1.5 (*n:n*), 1:2 (*n:n*)). The permeation enhancer (Na-taurocholate), the local pH regulator (citric acid), and the cyclodextrin used as a taste masking agent were included in the PVP-based shell phase of the fibrous formulation.

Electrospinning experiments were summarized in 2-factor, 3-level experimental designs. In the aqueous core precursor solution, the amount of active ingredient was determined to be 3% *w*/*w* (this parameter was kept constant during the experiments). The molar ratio of levocetirizine to HP-β-CD (factor 1) and the concentration of PVA (*w*/*w*%) (factor 2) were examined at 3 levels (−1, 0, +1). The applied parameters of the independent variables were determined based on lengthy preliminary experiments (Table 1).

For pure aqueous shell precursor solutions, the amount of sweet-tasting cyclodextrin (HP-β-CD) was kept constant at 10% *w*/*w*. The amount of sodium taurocholate was determined to be 0.1% *w*/*w* based on literature data. During the experiments, the concentrations of citric acid and polymer (PVP) were examined at 3-3 levels (Table 2).

For the coaxial spinning experiments, the solutions that proved to be the most suitable for single needle spinning were used. In the first experiments, pure aqueous solutions were electrospun. Later, however, the amount of ethanol in the shell solution was increased to promote the formation of a core–shell structure. We took advantage of the fact that PVA is not soluble in ethanol, so we hypothesized that in this way, it would be less able to mix the core and shell solution in the polymer drop at the end of the emitter. Water/ethanol mixtures of 10:0, 8:2, and 5:5 (m:m) were also tested in the shell precursor.

#### 2.2.2. Electrospinning Process

A laboratory-sized coaxial electrospinning device (SpinSplit Kft., Budapest, Hungary) was used to prepare the fibrous samples. Homogeneous precursor solutions placed in a plastic syringe (Luer lock syringe, Sigma Aldrich Ltd., Budapest, Hungary) were connected to a conventional emitter (22 G) and a coaxial emitter (22–18 G core–shell) with a Teflon tube. Pumps ensured even dosing of the solutions. The voltage varied between 10 and 25 kV during fiber formation, while the emitter–collector distance was varied between 10 and 20 cm. The spinning experiments were performed in a well-tempered room with a temperature of 22 ± 1 °C and 40 ± 5% humidity.

### 2.3. Morphologycal Characterization of Fibrous Samples

The morphological characterization of the electrospun samples was performed with a JEOL JSM-6380LA type scanning electron microscope (SEM). Measurements were performed at an accelerating voltage of 15 kV and a sample distance of 10 mm. Samples formed on the aluminum foil were attached to copper ingots with double-sided carbon adhesive and then examined after gilding at multiple magnifications.

### 2.4. Solid-State Characterization Methods

The fibrous samples and their components were analyzed with a Horiba Jobin Yvon Labram Raman microspectrometer. The excitation laser was a Nd-YAG laser radiating at 532 nm, which caused a change in the vibrational energy states of the molecules. The laser light was delivered to the samples by an Olympus BX-41 light microscope. For the reference samples, a magnification of 50x was used, while for the fibrous samples, a magnification of 100x was used for focusing. Raman-scattered photons were detected by a CCD detector after reflection. Measurements were performed in the 346–1789 cm^−1^ range, which carried most of the Raman signals. It was only necessary to reduce the intensity of light at a nominal 40 mW with a filter for the pure drug. A D0.6 optical filter was used for levocetirizine only to prevent degradation. It is important to note that the active ingredient in the fibers was protected from degradation by HP-β-CD and PVA. To achieve the appropriate signal-to-noise ratio, spectra were recorded from the reference samples in 10–90 s. The PVP and PVA polymers used as matrix material in the fibrous samples showed clearly distinguishable signals, and the signs of the HP-β-CD complexing material also appeared strongly. The peaks of the drug, in particular at 998 and 1600 cm^−1^, were very intense, which may therefore also be suitable for detection in drug-containing fibers. Spectra were generated from the fibrous samples at 90 s. To evaluate the spectra of samples prepared by coaxial electrospinning, spectra were recorded from fibers formed from core and shell constituents, respectively.

### 2.5. In Vitro Drug Release Study and Kinetic Modelling

The dissolution test was performed by further miniaturization of the method previously developed by the group. Our goal was to develop a method that brings oral conditions closer, primarily in terms of volume. Therefore, the dissolution test was performed in a reduced volume of 10 mL, which better demonstrates the oral conditions than the dissolution test methods found in the pharmacopeia. Dissolution was performed in a beaker (inner diameter: 23 mm), and the temperature and mixing of the dissolved drug in the dissolution medium were ensured with a temperature-controlled magnetic stirrer. The temperature of the dissolution medium was 37 ± 0.5 °C, and the stirring rate was 100 rpm. The method was developed by analogy with the swivel basket method found in Pharmacopoeia (Ph.Hg. VIII). The nanofiber samples removed from the collector were wound on a magnetic stirrer and then placed in a 4 mm diameter steel coil to ensure that the samples would sink to the bottom of the beaker and be fully immersed in the dissolution medium. An in-line probe of a Jasco-V-750 UV-VIS spectrophotometer was immersed in a pH 6.8 phosphate-buffered medium, and the dissolution of the drug was monitored for 5 min. The amount of drug dissolved was monitored at 231 nm for levocetirizine. The amount of dissolved drug was determined by a partially validated spectrophotometric method based on a preliminary calibration. Three parallel measurements were made from each composition.

For the evaluation of the kinetics of drug release, the Weibull model was fitted to the dissolution curves as follows:

(1) $$M_{t}=M_{\infty }\left(1-e^{-\frac{{\left(t-t_{0}\right)}^{\beta }}{\tau_{d}}}\right)$$

where *M_t_* is the amount of drug released up to time *t* and *M_∞_* is the maximum amount of drug released. The shape parameter of the function is denoted by *β*, the lag time by *t*_0_, and the mean dissolution time (where 63.2% of the drug is dissolved) by *τ_d_*.

The shape parameter (*β*) of the curve provides information on the time distribution of the dissolved drug. *β* = 1 indicates first-order kinetics, *β* > 1 shows a slow onset and then an accelerating drug release, and *β* < 1 shows a fast onset and a decelerating drug release.

The Microsoft Excel 2010 solver function was used in the nonlinear parameter estimation.

### 2.6. In Vitro Drug Release Study and Kinetic Modelling

The taste masking properties of the formulated complex nanostructure were tested by the Alpha ASTREE II potentiometric electronic tongue (Alpha M.O.S., Toulouse, France). The electronic tongue (e-tongue) was equipped with a sensor array (#2) including seven CHEMFET (chemically modified field-effect transistor) sensors developed for pharma applications (Alpha MOS, 2009). The electrode potentials of the seven sensors were recorded versus the reference electrode (Ag/AgCl 3M KCl) (Metrohm AG). There were five samples involved in the e-tongue tests: (1) the formulated complex nanostructure without API (Placebo), (2) the formulated complex nanostructure with a 50% dose of the API (Form+API_50%), (3) the formulated complex nanostructure with the total dose of the API (Form+API_100%), (4) the API, and (5) the Cetirizin Hexal (Commercial).

The e-tongue tests were performed under constant room temperature (25 ± 0.5 °C) using a random sampling order. Distilled water was used between the measurement of the samples for the cleaning of the sensors. Each sample was measured nine times using the following test conditions: 100 mL sample volume, 120 s acquisition time of the samples, and 20 s for the cleaning process.

The average value of the last 10 s of every reading and of every sensor was calculated and used for further statistical evaluation. Results of the e-tongue tests were evaluated by principal component analysis (PCA) to detect outliers and to visualize the relative taste differences measured by the sensors of the e-tongue. The statistical evaluation was performed by the R-project statistical software Ver. 4.1.0 (R Core Team, Vienna, Austria).

## 3. Results and Discussion

### 3.1. Morphology Study of the Electrospun Samples

Morphological characterization of the electrospun samples from the core and shell precursor solutions with a single needle electrospinning setup was carried out with SEM.

**Figure 1:**
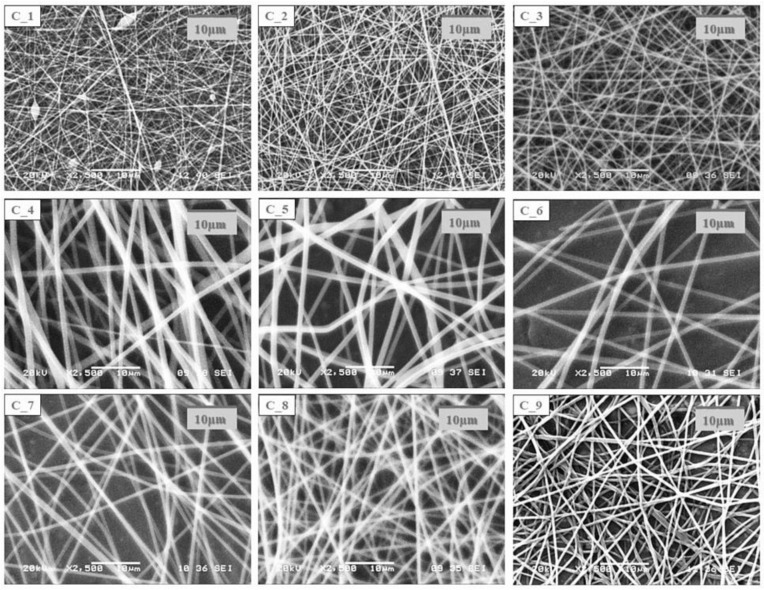
SEM images of electrospun samples prepared from different composition core precursor solutions (magnification: 2500×).

**Figure 2:**
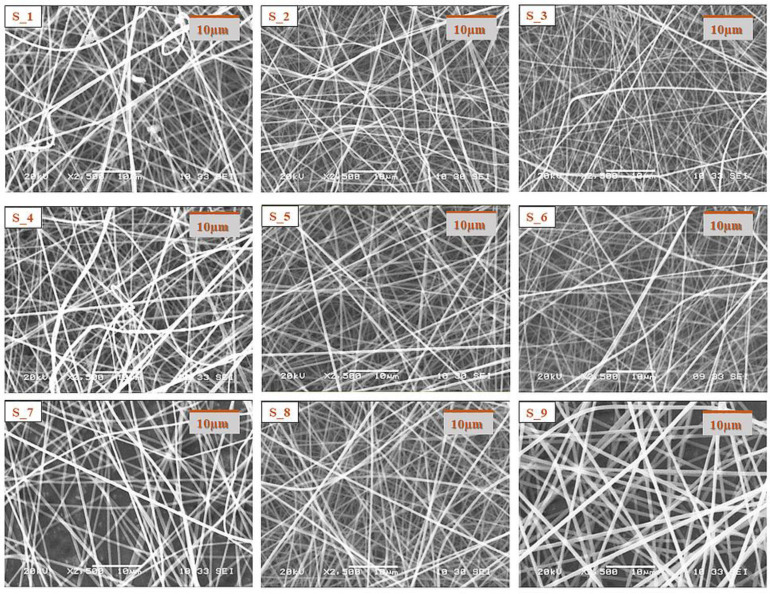
SEM images of electrospun samples prepared from different composition shell precursor solutions (magnification: 2500×).

The SEM images of the electrospun samples (Figure 1 and Figure 2) showed that in the case of most compositions, a clearly fibrous structure was formed. The only exception was sample C1, where a bead-like structure appeared to a small extent. In the case of the solution of the lowest CD and polymer combination, bead formation can also be observed; however, the structure was fibrous. The increasing CD and/or polymer concentration had a positive effect on the fiber formation. During the design of the final formulation, the amount of active substance in the fibrous product should be taken into account. As the drug content of the core precursor solution was constant, with the increasing amount of the excipient, the drug content of the fibrous product decreased; thus, the size of the final product should be increased. The latter has an impact on the formulation because of the anatomy of the oral cavity. On the other hand, the increasing CD amount can be beneficial for taste masking.

In all cases of the electrospinning of shell precursor solutions, a pure fibrous structure was obtained. The choice of the polymer solution used for coaxial fiber formation was determined by the dry matter content of the fibrous sample.

Based on the above for the coaxial electrospinning experiments, the precursor solutions found to be the most suitable for SNES were used. In the first coaxial electrospinning experiments, fiber fabrication was performed from pure aqueous precursor solutions (C3 and S3 precursor solutions). However, later, the amount of ethanol in the shell solution was increased (water/ethanol = 10:0, 8:2, 5:5 mass ratios were used) in order to promote the formation of the core–shell structure. Based on the camera recording of the electrospinning apparatus, the 8:2 water/ethanol mass ratio was found to be the most suitable.

**Figure 3:**
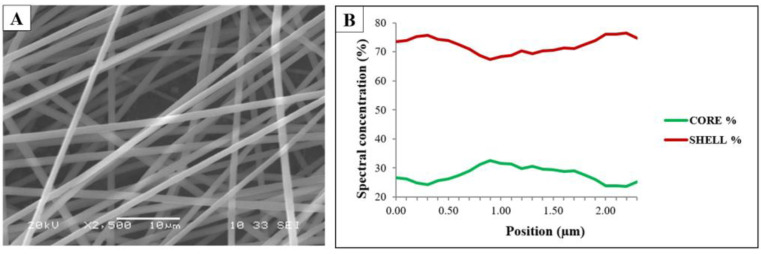
SEM image of the coaxially electrospun sample (**A**) and the result of the Raman mapping of the coaxially electrospun sample (**B**).

The coaxially electrospun samples were characterized using SEM (Figure 3A) and the fiber structure was investigated by Raman spectroscopy (Figure 3B).

The sample was found to have a clearly fibrous structure without beads and film-like elements. The Raman spectroscopy measurements were used to investigate the core–shell structure of the coaxially electrospun fibrous samples. The Raman maps (Figure 3B) show the contributions of PVA and PVP in the prepared sample. The green line represents the spectral concentration of the PVA (core polymer), while the red line represents that of the PVP (shell polymer). The signals from the edge of the fiber mainly came from the shell polymer, but not only from the shell. The signals from the center part included contributions from both the polymers.

The results of the Raman spectroscopy measurement indicated that as a result of the coaxial electrospinning the desired core–shell structure was not formed completely, and somewhat mixed fibers were achieved, which could be attributed to the slight mixing of the two precursor solutions, indicating that the interfacial stability between the inner and outer solution was not perfect.

### 3.2. Solid-State Characterization of the Fibrous Samples

**Figure 4:**
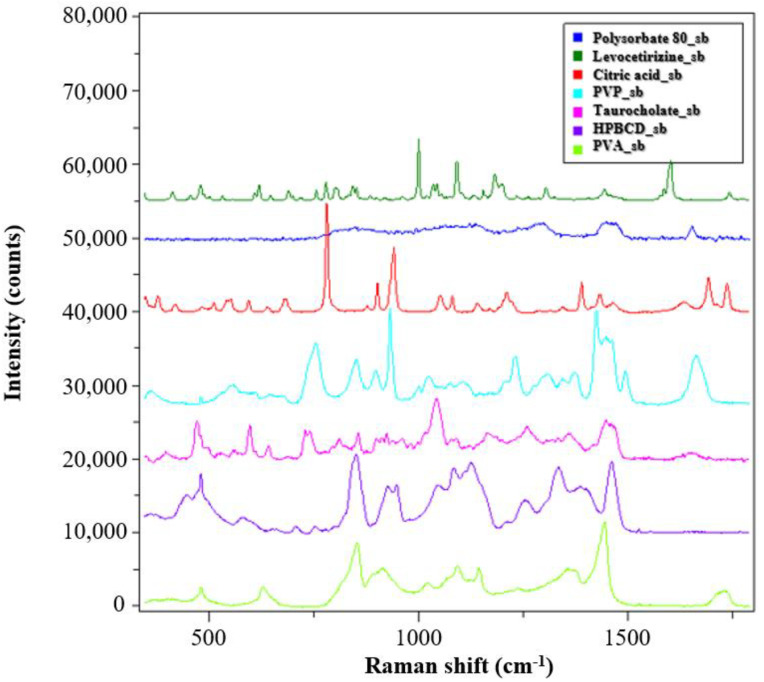
Raman spectra of the reference materials used for the fiber formation process.

The solid-state characterization of the samples was carried out by Raman spectroscopy. The spectra of the reference materials are shown in Figure 4.

The PVP and PVA polymers used as matrix material in the monolithic and core–shell fibrous samples showed clearly distinguishable signals, and the signs of the HPBCD complexing material also intensively appeared. The peaks of the levocetirizine at 998 and 1600 cm^−1^ were very intense, which may make them suitable for detection in the drug-loaded fibers.

**Figure 5:**
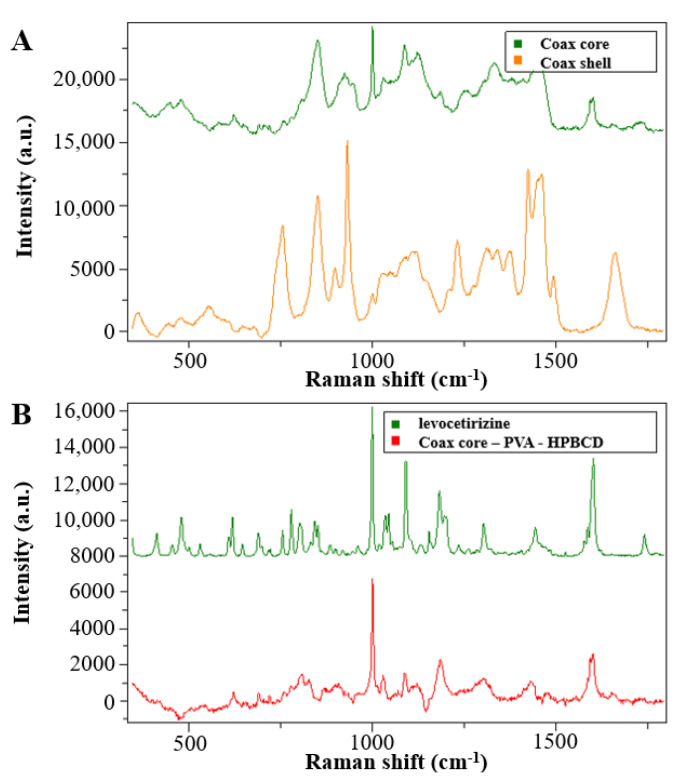
Raman spectra of the coaxially electrospun samples (**A**) and the crystalline levocetirizine and its embedded form (**B**).

For the evaluation of the spectra of the coaxially electrospun fibers, spectra were recorded from the monolithic fibers prepared from core and shell precursor solution (Figure 5A). In the Figure 5B spectra of the coaxially electrospun fibrous sample, it can be seen that, in addition to the main components, the signals of the active pharmaceutical ingredients also appeared. To better illustrate the drug signals in the fiber, the signals of the PVA and HPBCD signals were subtracted from the spectra of the electrospun sample. The residual spectrum (Figure 5B, red line) showed a significant change compared to the crystalline drug.

The characteristic Raman signals were merged and broadened, which is a consequence of amorphization. Thus, it can be concluded that as the result of the fiber formation, an amorphous solid dispersion was formed.

### 3.3. In Vitro Drug Release from the Nanofibers and Kinetic Study

**Figure 6:**
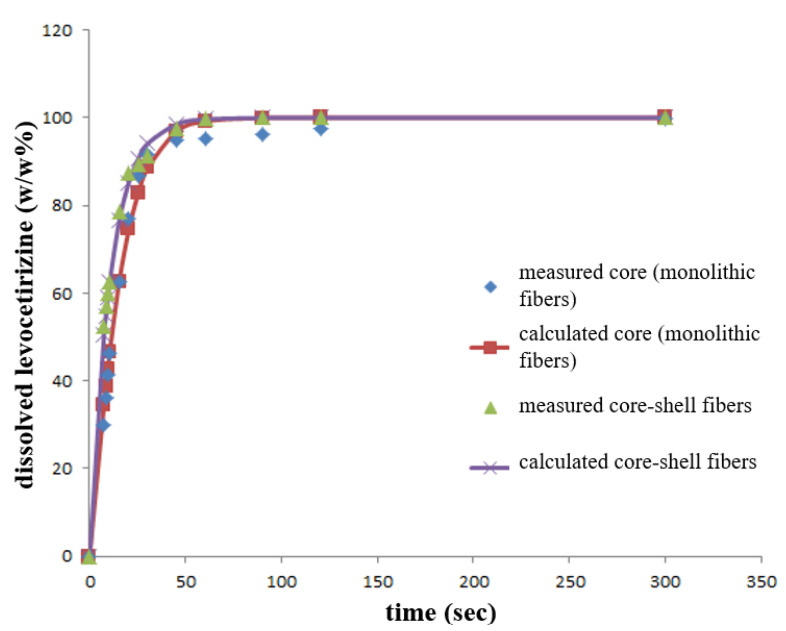
Dissolution profile of levocetirizine-containing monolithic fibers prepared by electrospinning, the coaxially electrospun core–shell fibers (these are the measured curves), and the corresponding calculated curves based on the Weibull model.

The results of the dissolution test performed in a reduced dissolution medium volume are shown in Figure 6. Fast levocetirizine release was obtained from the monolithic and also from the complex structural fibrous system; within 1 min complete dissolution can be observed. This is due to the increased surface area to volume ratio and porous structure of the nanofibers together with more favorable wettability properties, which enable immediate dissolution.

The release for each formulation was characterized by a saturation curve, starting with an initial fast release and reaching 100% after 2 min, corresponding to an immediate release formulation. In this case, the Weibull model can be used to characterize the kinetics of release. When this model is used, the shape parameter of the curve (β) can provide information on the time distribution of the released drug (β = 1 indicates first order kinetics, β > 1 indicates a slow onset followed by an accelerated release, and β < 1 indicates a fast onset followed by a slow release). The dissolution kinetic parameters of the different samples are summarized in Table 3. The release profiles were fitted well to the Weibull model. As can be seen in Table 3, the β values are similar, all around 1, which refers to a drug release mechanism consisting of swelling, erosion, and diffusion [31,32,33,34]. Based on the dissolution results, the release kinetics of levocetirizine from the complex core–shell nanostructure did not show a remarkable difference compared to the homogeneous core structure nanofiber, and with good correlation were following first-order kinetics, resulting in rapid and complete drug release.

### 3.4. Results of the Taste Masking Characterization of the Formulated Complex Nanostructure with the Electronic Tongue

**Figure 7:**
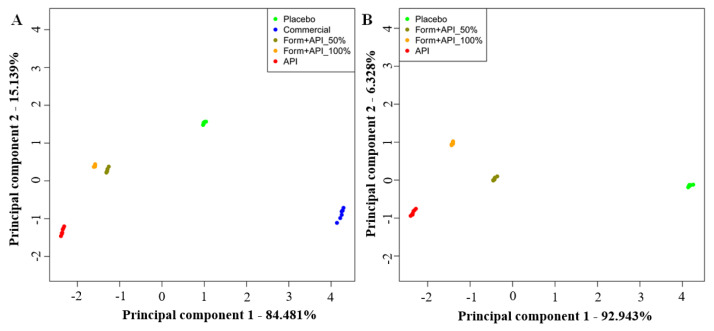
Principal component (PC) analysis score plot of the results of the e-tongue tests presenting the taste profile comparison of (**A**) all the five tested samples and (**B**) without the Commercial sample.

Results of the e-tongue tests performed to determine the taste masking efficiency of the formulated complex nanostructure against the API expressing an unpleasant bitter taste are presented in Figure 7. The PCA score plots show the relative taste profile comparison of all the five tested samples (Figure 7A) and without the Commercial sample (Figure 7B) after leaving out the results of the first two repeats of the experiments, which were found to be outliers.

The relative taste profile comparison of all the five tested samples (Figure 7A) clearly shows the best separation of the data points of the Commercial sample from the groups of the other four samples based on the first principal component (PC1). The group of the Placebo sample presented the second-best separation from the points of the API sample group followed by the groups of the formulated complex nanostructure with a 50% (Form+API_50%) and total dose of API (Form+API_100%), respectively.

Results of PCA calculated without the data of the Commercial sample confirmed the clear taste differences of the API sample and the samples of the formulated complex nanostructure with different API doses measured by the e-tongue (Figure 7B). The data points of both the formulated complex nanostructures containing API (Form+API_50% and Form+API_100%) presented a significant separation from the group of the API sample and the group of the Placebo sample not including API. These results confirm the taste masking characteristic of the formulated complex nanostructure.

## 4. Conclusions

The presented usage and utilization of coaxial fiber formation containing various enabling excipients in addition to the API in one system is novel, and no antecedents can be found in the literature. Furthermore, the core–shell type complex nanofibrous structure enabled fast and complete drug release with an acceptable taste masking effect, which could be a promising alternative to assure patient-centric rapid drug dissolution and consequent absorption without taking water. Since electrospinning is a continuous fiber formulation technique, the scaling-up of the process enables industrial feasibility, thus providing a value-added alternative for other immediate-release products available on the market.

## Figures and Tables

**Table 1:** Two factorial design table of the composition of precursor solution used for electrospinning experiments, which were then used as a core precursor solution in the coaxial fiber formation process.

Core Precursor Solutions		Factor 1: (Drug: HP-β-CD Molartio) ^1^		
		*Level* −1: 1:1 (*n:n*)	*Level* 0: 1:1.5 (*n:n*)	*Level* +1: 1:2 (*n:n*)
**Factor 2:** **(c _PVA_ *w*/*w*%) ^1^**	***Level* −1**: 10%	C_1	C_2	C_3
	***Level* 0**: 11%	C_4	C_5	C_6
	***Level* +1**: 12%	C_7	C_8	C_9

^1^ Factor 1: levocetirizine and HP-β-CD mol ratio and Factor 2: PVA concentration (*w*/*w*%). (The concentration of the levocetirizine (drug) was kept constant during the experiments.)

**Table 2:** Two factorial design table of the composition of precursor solution used for electrospinning experiments, which were then used as a shell precursor solution in the coaxial fiber formation process.

Shell Precursor Solutions		Factor 1: c _citric acid_ (*w*/*w*%) ^2^		
		*Level* −1: 0.1%	*Level* 0: 0.2%	*Level* +1: 0.3%
**Factor 2:** **c _PVP_ (*w*/*w*%) ^2^**	***Level* −1**: 14%	S_1	S_2	S_3
	***Level* 0**: 15%	S_4	S_5	S_6
	***Level* +1**: 16%	S_7	S_8	S_9

^2^ Factor 1: citric acid concentration (*w*/*w*%) and Factor 2: PVP concentration (*w*/*w*%).

**Table 3:** Dissolution kinetic parameters of levocetirizine-containing monolithic fibers prepared by electrospinning and the coaxially electrospun core–shell fibers.

Sample	*β* Parameter	*τ_d_* (s)	*M*_∞_ (%)	Correlation Coefficient
**Core monolithic fibers**	1.1621	15.5922	99.9	0.9949
	1.0281	14.22118	99.9	0.9908
	1.0069	20.686	100.0	0.9918
**Core–shell fibers**	0.9199	5.8	99.9	0.8539
	0.9913	10.6	100.0	0.9988
	1.0837	10.5	99.46	0.9981

## Data Availability

Not applicable.
